# Supplementary material for: Qualitative and Quantitative Analysis of Ukrainian Iris Species: A Fresh Look on Their Antioxidant Content and Biological Activities
Source: Molecules. 2020 Oct 8;25(19):4588. doi: 10.3390/molecules25194588 (PMC7582944; doi:10.3390/molecules25194588)
Supplement: Supplementary file 1 [file molecules-25-04588-s001.pdf]

SUPPLEMENTARY MATERIAL

Qualitative and quantitative analysis of Ukrainian Iris species: A fresh look on their content and biological activities

Olha Mykhailenko†, Michal Korinek†, Ivan Bezruk, Liudas Ivanauskas, Artem Myhal, Vilma Petrikaitė, Mohamed El-Shazly, Guan-Hua Lin, Chia-Yi Lin, Chia-Hung Yen, Bing-Hung Chen, Victoriya Georgiyants* and Tsong-Long Hwang*

Table of Contents

Figure S1. The UPLC-MS/MS-chromatogram of the methanolic extract *Iris hungarica* rhizomes 2

Figure S2. The UPLC-MS/MS-chromatogram of the methanolic extract of *Iris variegata* rhizomes 2

Figure S3. The UPLC-MS/MS-chromatogram of the methanolic extract of *Iris pallida* rhizomes 3

Figure S4. The UPLC-MS/MS-chromatogram of the methanolic extract of *Iris sibirica* rhizome 3

Table S1. The specificity of eleven quantified compounds and their chemical structures.... 4

Materials and Methods 5

References 8

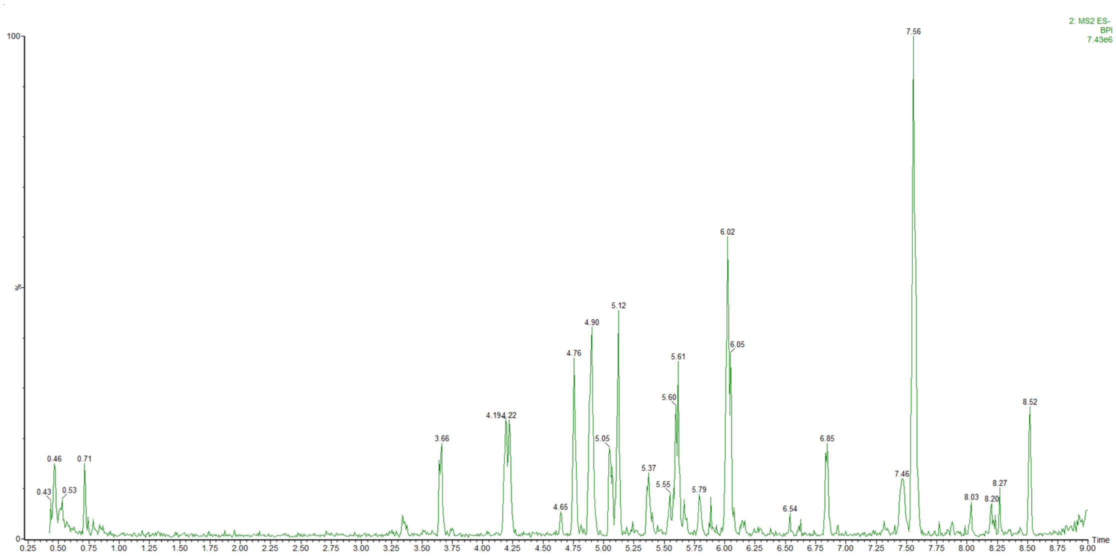

Figure S1. The UPLC-MS/MS-chromatogram of the methanolic extract *Iris hungarica* rhizomes

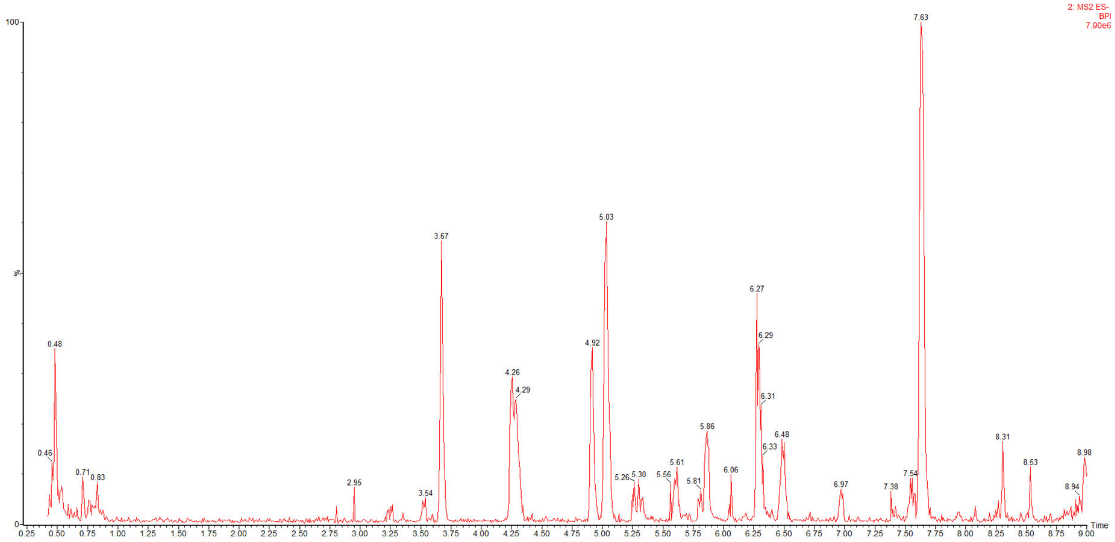

Figure S2. The UPLC-MS/MS-chromatogram of the methanolic extract of *Iris variegata* rhizomes

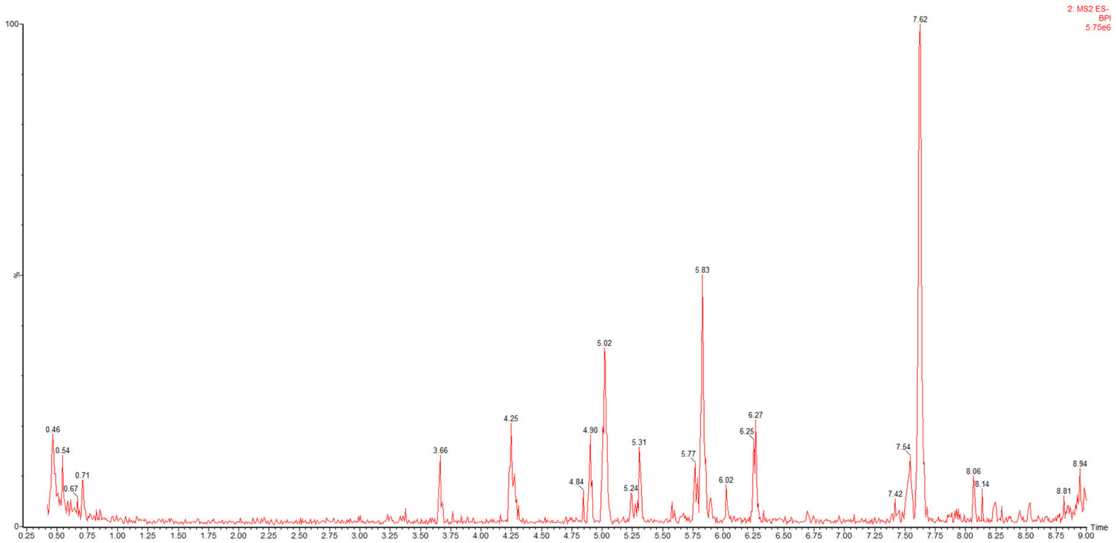

Figure S3. The UPLC-MS/MS-chromatogram of the methanolic extract of *Iris pallida* rhizomes

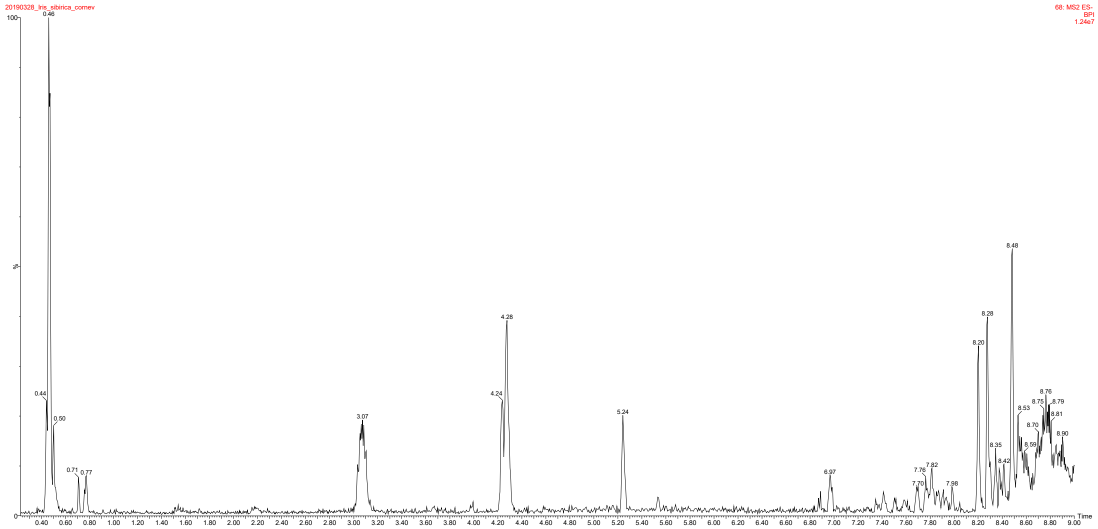

Figure S4. The UPLC-MS/MS-chromatogram of the methanolic extract of *Iris sibirica* rhizome

Table S1. The specificity of eleven quantified compounds and their chemical structures.

Mangiferin

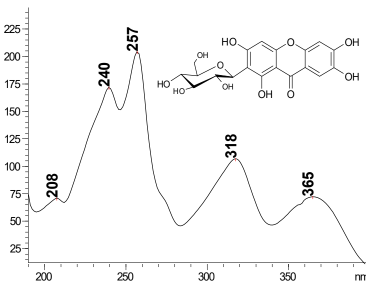

Germanaism B

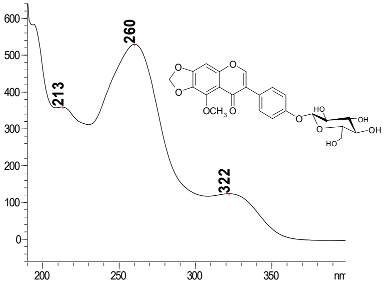

Irisolidone-D-glucoside

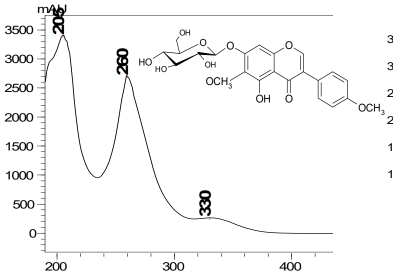

Irisolidone

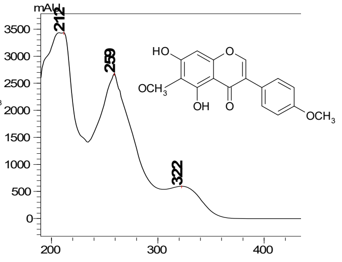

Tectoridin

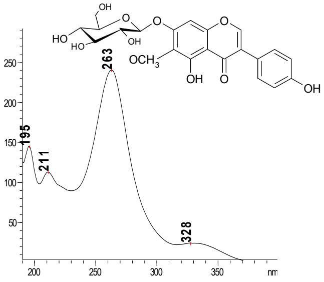

Iristectorigenin B

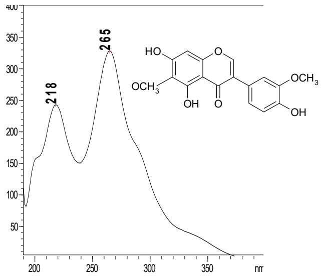

Irisolone (Nigricin)

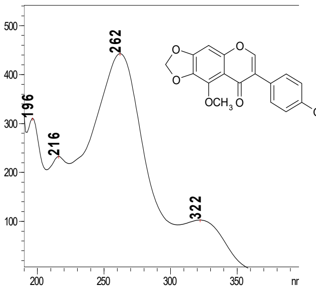

Irigenin

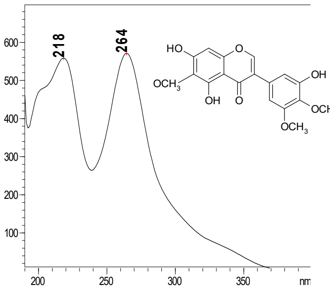

5,6-Dihydroxy-7,8,3′,5′-tetrametoxyisoflavone

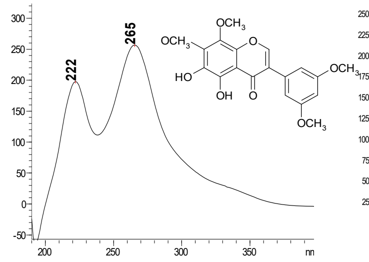

Gallic acid

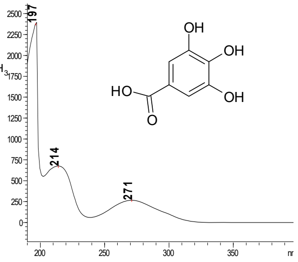

Caffeic acid

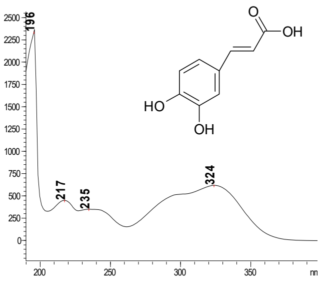

Materials and Methods

Details of procedures from Section 3 of main text are described below, including HPLC methodology and bioactivity assays.

3.9. HPLC-PDA conditions and HPLC post-column assay

Waters Alliance 2695 (Waters, Milford, USA) separation module system equipped with Waters 2487 UV/VIS and Waters 996 PDA diode-array detector (DAD) were used. HPLC grade water was obtained from a water purifying system (Millipore, Bedford, MA, USA). 2,2′-Azino-bis (3-ethylbenzothiazoline-6-sulfonic acid) diammonium salt (ABTS, purity ≥ 98%), potassium persulphate (purity ≥ 99 %) were purchased from Sigma (St. Louis, MO, USA), and Trolox (purity ≥ 98%) from Fluka Chemika (Buchs, Switzerland). The compounds separation was carried out utilizing an ACE C18 column (250 mm × 4.6 mm, 5.0 µm, Pennsylvania, USA) with a YMC guard cartridge ODS-A (3.0 µm, 10 mm × 4.0 mm). The binary solvent system of the mobile phase comprised solvent A (0.1% acetic acid in water) and solvent B (acetonitrile). A following linear gradient program was applied: 0–8 min, 5–15% B; 8–30 min, 15–20% B; 30–48 min, 20–40% B; 48–58 min, 40–50% B; 58–65 min, 50%; 65–66 min, 50–95% B. The injection volume of the sample solution was 20 µL. After PDA detection, the ABTS solution was mixed with the mobile phase carrying the analytes in the reaction coil (Raudonis et al., 2009; Raudonis et al., 2012). Empower Software Chromatographic Manager System (Waters Corporation, Milford, USA) was used to analyze the data. The ABTS post-column chromatograms were detected at the wavelength of 650 nm using Waters 2487 UV/VIS detector (Waters Corporation). The standard antioxidant Trolox (0.3995 µmol/g) was used for the preparation of the calibration curves (Marksa et al., 2016). Trolox equivalent antioxidant capacity (TEAC) was used to express antioxidant activity. The value was calculated as µmol Trolox equivalent (TE) for 1 g of dry mass (DM) of the plant material using the following formula: TEAC = c × V/m (µmol/g) where ‘c’ is the Trolox concentration in µM established from the calibration curve, ‘V’ is the plant material extract volume in L, and ‘m’ is the weight (precise) in g.

3.12. In-vitro assessment of NRF2 activity

The cell line HaCaT/ARE was developed from a HaCaT stable cell line carrying a fragment derived from pGL4.37[luc2P/ARE/Hygro] plasmid that contains four copies of an antioxidant response element (ARE) that drives the transcription of the luciferase reporter gene luc2P. The reporter cells were cultured in Dulbecco’s Modified Eagle’s Medium (DMEM) (Gibco BRL, Grand Island, NY, USA) supplemented with penicillin (100 U/mL), streptomycin (100 µg/mL), 10% heat-inactivated fetal bovine serum (HyClone, Logan, UT, USA), and 100 µg/mL hygromycin. The cells were maintained in a humidified incubator with 5% CO_2. Reporter cells were seeded (1×10^4 cells/well) in 96-wells plate, then were treated with the indicated concentration of the sample for 18 h (single measurement). Resazurin (Cayman Chemical, Ann Arbor, MI, USA) with a final concentration of 0.1 mg/mL was added and the cells were incubated for an additional 4 h at 37 °C. Fluorescence of the reduced resazurin in the supernatant of the cell (ex/em: 530 nm/590 nm) was detected using a Synergy HT Multi-Mode Reader (BioTek, Winooski, VT, USA) to determine cell viability. The cells were then harvested and luciferase activity measurements were achieved according to the manufacturer’s

protocol (Promega Corporation, Madison, WI, USA). The luciferase activity was normalized to cell viability and the relative luciferase activity was calculated and compared with the control (DMSO).

3.13. Lipid droplet assay

Lipid droplet accumulation was established by treating Huh7 cells with BSA-conjugated oleic acid. Cells were seeded in µClear® 96-wells plates (Greiner Bio-ONE, Frickenhausen, Germany) and treated with oleic acid and the tested drugs or DMSO for 18 h. Paraformaldehyde was used to fix the cells, which were stained with 2 µg/mL Hoechst 33342 and 1 µg/mL BODIPY® 493/503 HCS instrument was used to take and analyze images of the nuclei and lipid droplets (ImageXpress Micro System, Molecular Devices, Sunnyvale, CA, USA). The diameter settings were 8–25 µm for the nuclei and 0.5–2 µm for the lipid droplets.

3.14. In vitro assessment of the anti-allergic activity

3.14.1. Chemicals and reagents

Dulbecco's modified Eagle's medium-high glucose powder (DMEM), [3-(4,5-dimethylthiazol-2-yl)-2,5-diphenyltetrazolium bromide] (MTT), *p*-nitrophenyl-*N*-acetyl-D-glucosaminide (p-NAG), penicillin and streptomycin, dexamethasone, calcium ionophore A23187, mouse anti-DNP IgE antibody, and dimethyl sulfoxide (DMSO) were purchased from Sigma-Aldrich (St. Louis, MO, USA). Moreover, fetal bovine serum (FBS) was obtained from Hyclone (Logan, UT, USA). Dinitrophenyl-conjugated bovine serum albumin (DNP-BSA) was purchased from Merck (Kenilworth, NJ, USA). All other chemicals and reagents were purchased at the highest possible purity and quality.

3.14.2. Cell Culture

The mucosal mast cell-derived rat basophilic leukemia (RBL-2H3) cell line was purchased from the American Type Culture Collection. Cells were grown in DMEM medium supplemented with 10% FBS and 100 U/mL penicillin plus 100 µg/mL streptomycin. Cells were cultured in 10 cm cell culture dishes (Cellstar) at 37 °C in a humidified chamber with 5% CO_2 in the air.

3.14.3. Cell viability assay

The degree of cell viability of each sample was calculated as the percentage of the control value (untreated cells). All experiments were repeated three times. The maximally tolerated dose of DMSO was 0.5%. It served as control not affecting RBL-2H3 cell growth. Triton X-100 (0.5% solution) was used as the positive control causing the death of all cells in a well.

3.14.4. Degranulation β-hexosaminidase assay induced by A23187 and antigen

RBL-2H3 cells were dispensed into the 96-wells plate at a density of 2×10^4 cells/well (A23187-induced assay) or 48-wells plate at a density of 3×10^4 cells/well (antigen-induced assay). Cells were incubated at 37 °C in 5% CO_2 for at least 5 h to allow the cells to completely adhere to the bottom of the wells. Cells were washed with PBS and various concentrations of the samples or medium (untreated control) were added to each well (100 µL), followed by 20

h of incubation at 37 °C in 5% CO_2. Dexamethasone (10 nM) was used as a positive control. The cells for antigen-induced assay were sensitized with anti-DNP IgE (0.1 μg/mL) for 2 h. Afterwards, the cells were washed by pre-warmed Tyrode's buffer (135 mM NaCl, 5 mM KCl, 1.8 mM $CaCl_2$, 1.0 mM $MgCl_2$, 5.6 mM glucose, 20 mM HEPES, and 1 mg/mL BSA at pH 7.4) and were stimulated by calcium ionophore A23187 (1 μM) or cross-linking antigen DNP-BSA (100 ng/mL) diluted in Tyrode's buffer. The cells were incubated at 37 °C in 5% CO_2 for 1 h. Unstimulated cells were either lysed with 0.5% Triton X-100 solution for the total amount of *β*-hexosaminidase release or left untreated for the spontaneous release of *β*-hexosaminidase. Stimulated but untreated cells served as the control. Then aliquots of the supernatants (50 μL) collected from the control and experimental wells were incubated with an equal volume (50 μL) of 1 μM of *p*-NAG (*p*-nitrophenyl-*N*-acetyl-*β*-D-glucosaminide) prepared in 0.1 M citrate buffer (pH 4.5) serving as the substrate for the released *β*-hexosaminidase. After 1 h of incubation at 37 °C, the reaction was quenched by the addition of 100 μL of stop buffer (0.1 M $Na_2/NaHCO_3$, pH 10.0). Absorbance was measured at 405 nm on a microplate reader. The inhibition percentage of *β*-hexosaminidase release from RBL-2H3 cells was calculated as the percentage of the control value (untreated stimulated cells) using the following equation:

$$\text{Inhibition}\,(\%) = \left[1 - \frac{(\text{ODsample} - \text{ODspontaneous})}{(\text{ODcontrol} - \text{ODspontaneous})}\right] \times 100$$

3.15. In vitro assessment of the anti-inflammatory activity

3.15.1. Preparation of human neutrophils

Blood was taken from healthy human donors (20-35 years old) by venipuncture using a protocol approved by the institutional review board at Chang Gung Memorial Hospital. Neutrophils were isolated using a standard method as previously described (Boyum, 1968).

3.15.2. Measurement of superoxide generation

Neutrophils (6×10^5/mL) supplemented with 0.5 mg/mL ferricytochrome *c* and 1 mM Ca^{2+} were equilibrated at 37 °C for 2 min and then incubated with the tested compounds or DMSO (control) for 5 min. Genistein served as a positive control. Cells were activated with formyl-methionyl-leucyl-phenylalanine (fMLF, 100 nM)/cytochalasin B (CB, 1 μg/mL) for 10 min. The absorbance was continuously monitored at 550 nm in a double-beam, six-cell positioned spectrophotometer Hitachi U-3010 with constant stirring (Hitachi Inc., Tokyo, Japan). Calculations were based on the differences in absorbance with and without superoxide dismutase (SOD, 100 U/mL) divided by the extinction coefficient for the reduction of ferricytochrome *c* (ε = 21.1/mM/10 mm).

3.15.3. Measurement of elastase release

Neutrophils were equilibrated with MeO-Suc-Ala-Ala-Pro-Val-p-nitroanilide (100 μM), an elastase substrate, at 37 °C for 2 min and then incubated with the drugs for 5 min. Genistein served as a positive control. Cells were activated by 100 nM fMLF and 0.5 μg/mL CB, and

changes in the absorbance at 405 nm were continuously monitored to monitor the elastase release. The results were expressed as the percent of the initial rate of elastase release in the fMLF/CB-activated drug-free control system.

3.16. In vitro assessment of cytotoxic activity

3.16.1. Cell culture

Blood was taken from healthy human donors using a protocol approved by the Chang Gung Memorial Hospital review board. Neutrophils were isolated according to the standard procedure described before (Boyum, 1968). The inhibition of superoxide anion generation was measured by the reduction of ferricytochrome *c* as previously described (Yang et al., 2013). Elastase release representing degranulation from azurophilic granules was evaluated as described before (Hwang et al., 2009). Details can be found in Supporting information.

3.16. In vitro assessment of cytotoxic activity

3.16.1. Cell culture

Human melanoma cancer cell line IGR39 and human triple-negative breast cancer cell line MDA-MB-231 were obtained from the American Type Culture Collection (ATCC, Manassas, VA, USA). Cells were grown in DMEM Glutamax medium (Gibco, Carlsbad, CA, USA) containing 10% fetal bovine serum and 1% antibiotic mixture (10,000 U/mL penicillin and 10 mg/mL streptomycin; Gibco). All cells were incubated at 37 °C in a humidified atmosphere containing 5% CO_2.

3.16.2. Cell viability assay

IGR39 and MDA-MB-231 cells were seeded (3,000 and 5,000 cells/well, respectively) in 96-well plate and incubated overnight at 37 °C in a humidified atmosphere containing 5% CO_2. Cells were affected by various concentrations of the tested extracts (from 1 mg/mL to 31.25 µg/mL). As a background control, the only medium without cells was used and the medium with 0.4% ethanol served as the negative control. After 72 hours of incubation with the extracts, 20 µL of MTT (5 mg/ml) was added into each well and incubated for 4 hours under the same conditions. The supernatant was removed and 100 µL DMSO was added. The absorbance was measured at 570 nm and 630 nm, and EC_{50} (half-maximal effective concentration of a drug at which 50% of its maximum response is observed) values were calculated.

References

Boyum, A., 1968. Isolation of mononuclear cells and granulocytes from human blood. Isolation of monuclear cells by one centrifugation, and of granulocytes by combining centrifugation and sedimentation at 1 g. Scand. J. Clin. Lab. Invest. Suppl. 97, 77-89.

Marksa, M., Radusiene, J., Jakstas, V., Ivanauskas, L., Marksiene, R., 2016. Development of an HPLC post-column antioxidant assay for Solidago canadensis radical scavengers. Nat Prod Res 30, 536-543.

Raudonis, R., Jakstas, V., Burdulis, D., Benetis, R., Janulis, V., 2009. Investigation of contribution of individual constituents to antioxidant activity in herbal drugs using postcolumn HPLC method. Medicina-Lithuania 45, 382-394.
Raudonis, R., Raudone, L., Jakstas, V., Janulis, V., 2012. Comparative evaluation of post-column free radical scavenging and ferric reducing antioxidant power assays for screening of antioxidants in strawberries. J. Chromatogr. A 1233, 8-15.
